# Emotional Distress in the Relationship of Caregivers and Institutionalized Babies

**DOI:** 10.3389/fpsyt.2020.00266

**Published:** 2020-04-27

**Authors:** Rosemeire Aparecida do Nascimento, Mirian Akiko Furutani de Oliveira, Gláucia Rosana Guerra Benute, Andrea Nogueira Landmann, Fernanda Galo, Thames Waléria Borges

**Affiliations:** ^1^ Faculty of Psychology, São Camilo University Center, São Paulo, Brazil; ^2^ Division of Psychology, Hospital das Clínicas, University of São Paulo, São Paulo, Brazil; ^3^ INSERM-Methods and Cultures, CESP (Center for Research in Epidemiology and Population Health), Paris, France

**Keywords:** institutionalized children, early childhood, substitute maternal care, social caregiver, emotional distress

## Abstract

Institutionalization is an exceptional and temporary measure that occurs when there is a violation of rights; lasting until the family reintegration or, in the impossibility of this, the placement in a substitute family through adoption. Among the main reasons for institutionalization in Brazil are the financial difficulties, abandonment, domestic violence, drug addiction, homelessness of the responsible for the child, sexual abuse, and the loss of parents by death or imprisonment. Although children and adolescents have their rights assured when they are institutionalized, the care provided in these spaces does not include all their needs and demands, which may damage their affective-relational development. Maternal deprivation in the first years of life can be detrimental to the development of these children, if not provided by adequate substitute care. Therefore, to understand which place the institutionalized baby occupies in the imaginary of the social caregivers and, from this, how is established the relationship regarding the care, is of fundamental importance to assess and address the risk factors in child development at this stage of life and in situation of institutionalization. This is an exploratory and descriptive study, developed in a childcare institution, located in the city of São Paulo, Brazil, capable of accommodating up to 20 babies between 0 and 2 years old. Data collection was performed with nine employees, eight social caregivers and one general service assistant who work directly in the care of the institutionalized babies. Drawing-Story with Theme (DS-T) procedures were used. Qualitative analysis was based on Interpretative Phenomenological Analysis. The appreciation of the nine applied Drawing-Story procedures allowed the establishment of three discussion axes representations of baby, baby care, and early separation process. This study concluded that the difficulties that permeate the context of caregivers’ work are the high turnover of institutionalized children, as well as employees, the difficulty of dealing with processes of bonding and breaking bonds and no recognition of the profession. We highlight that the place that these babies occupy in the imaginary of these caregivers influences the bond they establish with the children hence the care offered to the babies. It is also noteworthy that these caregivers exhibited anguish and suffering from the reasons they believed led the children to be institutionalized.

## Introduction

### Institutionalization in Brazil

In Brazil, 61% or 32 million children and adolescents are under the multiple dimensions of poverty. Of these, only 6 million, although belonging to financially deprived families, have their rights guaranteed. The others, besides living in poverty, have one or more rights violated, being in a situation of multiple deprivation. The United Nations Children’s Fund (1) highlights that almost half of Brazilian children and adolescents are violated by one or more of their rights.

Brazilian law states that in situations of violation of the rights of children and adolescents, in which moderate protective measures have not been effective, if it is impossible for the family of origin to provide sufficient care and protection for their child, institutional care or substitute family as an exceptional and temporary judicial measure (2, 3). This institutionalization must last until family reintegration, without exceeding the 2-year period. If there is no possibility of returning to the original family, within this period, the child should be placed in a substitute family by adoption (3).

Among the main reasons for institutionalization are the financial difficulties of parents or guardians, abandonment by parents, domestic violence, substance abuse of parents or guardians, homelessness, death or imprisonment of those responsible and, intrafamily sexual abuse (4, 5). Of the 76,216 allegations of violation of the human rights of children and adolescents in Brazil during 2018, 36.3% referred to negligence; 24.4% to psychological violence; 20.35% to physical violence, and 11.22% to sexual violence (6).

Regarding the quality of the services provided by the Brazilian sheltering institutions, there is also the predominance of the assistance function and the fragility of the commitment to the developmental issues of childhood and adolescence (7, 8); which is the opposite that says the Statute of Child and Adolescent (2, 3).

Although institutionalization in Brazil is a legal measure for a full protection and guarantee of rights, sometimes the activities carried out in the sheltering institutions do not meet the needs and demands of institutionalized children and adolescents, and it is necessary to rethink those institutions as environments that allow the construction of positive identificatory references (9, 10). Among the main difficulties encountered are the high number of children per caregiver, the standardized care, the lack of planned activities and the shift changes of the caregivers, which promote a break at the end of each period, not allowing a continuous routine of care (10, 11).

In addition, there is the difficulty of social caregivers, also called social mothers, in elaborating and separating maternal desire and motherhood from the exercise of professional activity. In this sense, it is important for professionals to be aware to the fact that they are not the mothers of the institutionalized children (12).

Many social educators interpret institutionalization as “an act of unloving and inhumanity on the part of the mother’s institutionalized children” (13), which influences your relationships with these children by assigning them a certain place and contributing to their social exclusion and belief in parental abandonment and unlovingness. Such conception is strongly observed when the sheltering occurs at an early age and the caregivers find themselves facing the abandonment and fragility of the baby (14).

Current data from “Conselho Nacional de Justiça” (National Council of Justice - CNJ) indicate that 47,000 children and adolescents in Brazil are in a condition of institutionalization (15), and of these, it is estimated that only 5% are in a family shelter situation, in other words, they are living in a family environment, with substitute families accompanying them until they can cease the shelter measure. For children aged zero to two years, there are more than three thousand in institutionalization (15). Regarding early childhood, regardless of the reason that leads to institutionalization, there will always be a rupture in the mother-child relationship, due to the induced separation in an essential phase for psycho-affective and motor development (13, 16).

### Maternal Care

According to Bowlby (17), substitute care can be a way of trying to reduce the damage caused by the deprivation of relationship with the parents of institutionalized children. The author points out that “firstly, we have to recognize that separating a child under the age of three from the mother is a very serious thing, one that should only be undertaken for good and solid reasons and, when done, must be planned with great care” (17) (p.8). Though, the care offered by the institutions might not be entirely adequate, due to the specificities of the functioning of these services and the relationships that social educators establish with the children (10). Institutionalized babies often receive basic care but not affection, which can trigger difficulties in physical, mental and social interaction (18, 19).

Spitz (19) considers the relationship between the child and the mother, or who makes this function, the most important condition for the development, because its reciprocity makes the child able to gradually build a coherent image of his world, because it is a special interaction that gives the baby “an exclusive world that is his own, with a specific emotional climate. It is this cycle of action and reaction that enables the baby to gradually transform meaningless stimuli into meaningful signs” (19) (p.43). His studies focusing on the effects of maternal deprivation on institutionalized infants under reduced or nonexistent care show a significant delay in their behavioral development in relation to children living in a family. Although basic hygiene and food care is adequate in institutions, there are implications that can influence bonding, such as the large number of children for few social educators, leading to a lack of stimulation and care (19), which can also be observed in the Bowlby’s studies (17).

### Psychological and Cognitive Development

In the first year of life there is the maturation and unfolding of innate phylogenetic functions; the child develops resources that allow him to adapt to the world around him, becoming progressively more independent from the environment. The mother, or her substitute, should present the environment to the baby through the emotional care offered, which are essential aspects for a healthy development. This, which is the first object relationship, allows the child to develop as a social being and serves as a model for the other relationships along their growth and insertion in other environments of society. It is understood that in the first phase of life the neurophysiological development is premature. The baby does not have the full perception of his own body, so he finds no difference between his and his mother’s (20). The maternal function is essential for the child’s psychic organization and constitution as a subject. It can be said that it is from the psychological organization developed through the relationship with the mother, or with the caregiver, that the child gains the ability to relate to other human beings (21).

Thus, maternal deprivation has consequences for the establishment of the baby’s mental health (17, 19, 22) and might cause physical and mental distress, expressed through symptoms (23). Separation of the mother and the family environment at this stage of life can be considered an important traumatic experience.

According to Bowlby (24), attachment or bonding is developed in the baby as a result of the affective experiences shared with the environment in which he lives, especially in interaction with the mother figure. The attachment relationship is an instinctive behavior of the baby that consists of communication that intends to arouse the attention of the mother or caregiver.

The form of interaction between the mother or the representative of this function and the baby, the demonstration of interest in the care offered to the child, is what makes the fundamental bond for the child’s development (25), in other words, mothering of the baby is established from the affective bonds built through the care that the mother will give to her child. França (26) points out that the “main characteristic of children who have suffered lack or deprivation in this primordial bond is to have a very reduced capacity for adequate responses, both socially and emotionally” (p.9).

Considering that the child custody is taken out of the family and sent to an institution where social caregivers are the closest adults, it is important to think about the essential role they play with these institutionalized children (18).

However, often this professional practice is not recognized and valued, making them feel disposable. Although fundamental, especially in such an important period as early childhood, they see themselves as unimportant to the development of children. In addition, when a child leaves the institution, the bonding process usually breaks down and the caregiver is no longer part of the life of that child for whom had affection (11).

Long-term institutionalization favors transient attachment with various educators. Over time, the children begin to avoid any closeness, thus it is possible to observe the trauma caused by the loss and rupture of ties with those who perform the function of substitute maternal care (10).

Peiter (27) mentions that “the contact with different caregivers and the experience of discontinuities of bonds and unfinished separations can be much more complex” (p. 51), so that experiences of attachment and rupture in later relationships are experienced; as mourning processes.

The Romanian studies point out the importance of the emotional attachment and the impact of the institutionalization for the brain’s development (28).

Even with the consensus in studies on child development and protection that young children should be raised in families, that foster care in institutions can bring a lot of damage to their development (28), the Brazilian reality shows significant levels of risk and violation of the rights of children and adolescents, added to the low number of foster families that makes institutionalization still remains.

According to França (26), “It is possible for institutions to be organized in such a way that, despite the absence of the mother, the child can develop physically and psychically in a healthy way” (p.12). She cites the experience developed by Emmi Pikler, in Hungary, of institutional care for young children as a model to consider some necessary changes. It is important to note that although Pikler is known and respected worldwide; in Brazil her method is not applied in the field of institutionalization, but it is explored in the field of early childhood education, being used in early childhood education centers.

Pikler’s method is based on two essential conditions for the healthy development of babies: the establishment of a quality affective relationship; and the child’s free exploration of the world around him and himself, his movements, according to his interests and his pace (29–31).

Since birth, the child has resources to get in touch with the environment around him, which is, he has the necessary potential to actively contact his social and physical environment, but it will be his relationship with his mother, or whoever performs the maternal function, through his sensitive and adequate responses to the baby’s demands, his crying, his expressions, his movements, which will allow him to develop his real abilities (32).

The baby should not be taken as an object of manipulation for care, he is a subject, he needs the affective relationship with an adult caregiver, who will be his reference and a facilitator in his exploration of the world in a creative and autonomous way França (26). In the words of Falk (30) the act of caring must be taken as “an intimate moment, full of communication. The baby should not be considered as a simple object of care, but as a person who has an influence on events and establishes relationships, a true companion” (p. 34).

In Pikler’s method, in addition to providing spaces thinking about the baby’s need for him to move freely, it is also necessary to train professionals who take care of the babies in the institution, so that those professionals can understand the essentials of child development, as well as they became psychically available to the baby in their care (30, 31).

The bonding between social caregivers and institutionalized children can be considered an attachment relationship that, at times, is equivalent to the bonds of parents and children, and in this sense it is possible that the professionals who work in this context want to maintain the union with the child, often, causing suffering and frustration when the child is taken away (11). Thus, although the process of forming affective bonds is important and necessary, social caregivers cannot nurture the desire to take the place of a family member (20). In doing that, the position of social caregiver is permeated by the conflict between being affective and not being too attached.

Through the presence of caregivers, playing the role of social mother, institutions must be responsible not only for physical care, but also to provide support for the baby to have an appropriate psychic development. It is essential to offer the best possible care so that the child’s emotional development is stimulated, through the formation of attachment relationships, even in institutional settings (33, 34).

It is known that environments with few stimulation and inconsistent parenting can delay and impair the cognitive development in terms of global intellectual functioning and language (35). The relationship between the caregiver and the child, in a literature review study, proved to be a decisive factor for the good development of institutionalized children (36).

Studies by Rossetti-Ferreira, Serrano and Almeida (37); Carvalho (38); and Keller (39) point out the importance of observing the formation of bonds as “a process co-built in interactions and dialogical relationships located in different contexts” (34), as what happens in institutionalization.

It should be considered that the social caregiver of the institutionalized babies sometimes can receives information about how circumstances the institutionalization took place, for example, as a result of violence and neglect of care and, from this information, suffer from identification with the traumas suffered by those children. Lachal (40) considers that the contact of caregivers with the suffering of the babies can result in multifaceted and significant experiences. The author proposes that the fact that the babies are not able to express themselves verbally acts as a potentiator of countertransference reactions in those who take care of them. Postulates, moreover, that the baby reproduces the same relationship pattern that it experienced in situations of abuse or violence, tending to repeat, in relations with substitute caregivers, the same model of relationship established with those who previously attacked him. Therefore, attempts at new bonds would tend to suffer the interference of the same feelings that permeated the most initial relationships. The new encounter would therefore be susceptible to feelings of helplessness, fear, sadness, anger, etc.

Lachal (41), defines the moment of shared pact between the baby and the adult caregiver as a process of interaction between both, in which the adult caregiver enters the “world of the baby” sharing with him his perceptions, sensations and existing experiences. For the author, when we take care of babies in humanitarian situations and here, we also add the context of caring in public institutions for the care of babies who have experienced traumatic ruptures of early bonds, our countertransference can be complex and ambivalent. We must renounce a certain illusion that we are always maintaining a positive empathic relationship with the child. For Lachal (42), if we, those who give the care, the caregivers, do not consider this countertransferential dimension in the relationship of the care, we will lose some skills during the “care.” On the other way, by assuming the feelings of ambivalence in this countertransference process, we will be able to interact and better understand the baby’s world and its interactions. Who knows, we may even be able to “play” with the baby (42).

In this sense, this study aimed to understand what place the institutionalized baby occupies in the imaginary of social caregivers and how, from there, the relationship of care is established, which plays a fundamental importance to assess and face the risk factors in the child development at this stage of life and in a situation of institutionalization.

## Materials and Method

This study is part of a major research titled: “Assessment of risk factors for cognitive and affective development and early intervention in institutionalized babies from 0 to 2 years old.”

### Type of Research

This is a qualitative study of exploratory and descriptive characteristic, proposing institution is the São Camilo University Center, Psychology Department. The qualitative method aims to describe, understand and analyze the observed phenomena. In this method the knowledge emerges from a process of construction and reconstruction of the human being. It does not treat reality as objective, intrinsic and irreducible, but as construction on a permanent interdependent relationship between the subject, the object and the world (43).

Qualitative methods have historically been widely used in the human and social sciences and seek to describe a complex structure, to derive a theory, to produce hypotheses. Criteria that guarantee validity and credibility of the method are: the use of different sources of data collection; validation by the subject that is recognized in the description of the phenomenon; analysis by several researchers in order to strengthen the results achieved together; approach by a clearly defined theoretical field and the incorporation of sufficient perspectives to fully explore the phenomenon (44, 45).

### Research Location

The present study was conducted in a childcare sheltering institution located in the city of São Paulo, Brazil, with a maximum capacity to accommodate 20 babies between 0 and 2 years old.

### Sample

Data collection was performed with eight social caregivers and an assistant of general services who work directly in the care of the institutionalized babies.

About the participants, one is less than 20 years old, four are between 20 and 30 years old and four are between 40 and 50 years old. Six of the participants have children, three are not mothers and one expressed the desire to have a baby. All have completed high school, and none have university degrees.

To achieve the proposed objectives, the Drawing-Story procedure with theme (D-S with theme) derived from the Drawing-Story procedure designed by Trinca W. Trinca (46–49), was used. The Drawing-Story procedure was developed to be used as an auxiliary tool in psychological diagnosis. It is appropriate as a psychological resource to approach the mental world, allowing to identify fantasies, desires, anxieties, affections and feelings. A variation of the Drawing-Story procedure is the DS-T technique, which was developed by Vaisberg (50). It is an adaptation of the original procedure, facilitates the expression of subjectivity also allows the subjective investigation of any theme, which can be applied in different age groups, individually or in groups.

Such adaptation can favor emotional expression in a playful, relaxed, undefended way, allowing the development of research that includes different groups and social actors (46–49, 51).

It is a technique in which the examiner presents a blank sheet of paper, black pencil and colored pencil to the participant and asks him to make a drawing with the proposed theme. In this study, the proposed theme was “to draw a baby.” After the drawing was done, the examinee was asked to freely narrate stories about the baby drawn. Then, the researcher conducted a free inquiry, in order to clarify the main points of the story told, aiming at a better understanding of the participant’s psychic dynamics, the place that the baby occupied in his imagination, as well as the fantasies and expectations of taking care of a baby, and an institutionalized baby.

Such procedure, Drawing-Story, as Trinca (47) indicates when referring to Levy (52), accesses the expressive potential of drawing, allowing the elaboration of images and patterns, habits, emotions and attitudes, in a conscious way or not. The subject submitted to this procedure does not realize what he is expressing, because they are unconscious contents and, thus, will be less defensive (53).

Thus, it was decided not to use the semistructured interview instrument, seeking that social caregivers talk freely about babies and the care with them. Therefore, no type of interview was conducted, except the participants’ initial data, such as age, education, and whether they had children. From the first story reported, they were asked to tell more about the baby drawn, to deepen aspects of their narratives, such as what the baby was thinking or feeling, how the person responsible for the baby was thinking or feeling.

Trinca (49) points out that the DS-T procedure as a method of psychological investigation has been used in research aimed at elucidating the representations and understanding of the unconscious related to the social and collective context. It is a technique in which the examiner presents a blank sheet of paper, black pencil, and colored pencil to the subject and asks to draw with the proposed theme.

In this study, caretakers (social caregivers or other institution’s professionals) were asked to draw any baby and tell a story about him. From the first narrative brought by the participant, she was encouraged to speak freely about her perceptions about the baby drawn and the associations arising from them. The main themes addressed in their narratives were about the institutionalized children, motherhood, the family of the institutionalized children and the care of social caregivers.

### Ethics

The research was submitted and approved by the Ethics Committees of Centro Universitário São Camilo: Research Committee (CPq) Centro Universitário São Camilo-SP, Document number PQ.66/2018; and Embodied Opinion of CEP CAAEE 95842818.2.0000.0062, number 2.843.363.

All social caregivers and other professionals at the institution were aware and agreed with the study being conducted. After the explanation and the signing of the Free Consent Form, the projective procedure was initiated.

### Data Analysis

The data obtained in the drawings-story with theme followed what proposed by the Interpretative Phenomenological Analysis (IPA). It is a phenomenological method that aims to understand the meaning of behavior with a view of unity and totality, surpassing objective thinking. In this way, the researcher deepen into the phenomena of consciousness, starting from the analysis of the whole for the unit, understanding it from the point of reference of those who are part of the phenomenon that happens (54, 55).

In order to preserve the reliability of the research (44), the results were analyzed, discussed and compared by several researchers, ensuring that the definition of the themes found and derived from the analysis of the material obtained in the research, discarding the influence of the unique view of each researcher.

## Results

In this study, it was possible to verify three thematic axes: the first highlights the representations that caregivers have about the institutionalized baby; the second refers to the representations of caring for the baby and the third addresses the representations about suffering and the process of separation, suffering and trauma.

Each axis was divided into the found themes, as described in **Table 1**.

**Table 1:** Presentation of the axis and main themes.

Axes	Themes
Baby representation	Baby that stays/Baby that leaves Imaginary bay/Real baby Naming of the babies
The baby caregiving representation	Continuity and discontinuity in the bond and caring Motherin role/Biological mother—heart mother Limits in caring Frustration in caring—Ability to care for the baby/Ability to be with the baby Dyad of caring/group/institutional/social
Representation of separation and suffering	Survivor baby/rescued baby Suffering expressed by the body/Injured body Mistreated baby/untouchable baby Repetition of violence

### Axis 1: Representation of the Baby

It is the representation that the caregiver has about the institutionalized baby. It should be noted that the participating caregivers do not officially receive information about the baby’s history and the institutional care itself. From the reports of the technical team, the family or health professionals addressed to them, or conducted in their presence, they end up constituting a fragmented history of the baby. The Baby Representation axis consists of the following three themes:

#### Baby That Stays/Baby That Leaves

It is observed that, although the babies are in the shelter just temporary, the suffering of the caregivers is evident when the babies leave the institution. As can be seen in excerpts taken from the stories:

*“The babies in the shelter are like rain to me, they spend time and then they leave.”*

*“When she left, I felt sad because I couldn’t see her every day anymore[ … ]”*

*“I feel profound sadness to see that they are abandoned here, but we end up getting used to seeing so many stories and it takes a lot of strength because the children are distressed, and this passes on to us.”*

*“Before, I didn’t know how to deal with them leaving, I even got sick [ … ].”*

*“When they said he was going to be adopted, I cried (cries).”*

#### Imaginary Baby/Real Baby

The second axis contemplates the imaginary baby and the real baby. In the graphic representations, it was observed that all drawings represented older children.

Caregivers reported their wishes for a highly successful future for the babies they cared, in which they will assume the position of good caregivers:

*“I imagine her as a doctor, who comes to change things, does not do drugs, nor rebells”*

*“If he had left from this shelter, he would be a doctor, a doctor who would take care of me in the future, he would give love to others because in the shelter he received love and affection from us.”*

#### Naming of the Baby

All the caregivers spoke about the naming of the children:

*“I did the R.”*

*“I drew B.”*

*“The baby I drew has the same name as my son, it’s called V.”*

Some narratives related the names given to the babies who had already attended the shelter in the past, but to which they remained bonded, making the choice to represent them and tell about the care they had with them.

They seek for children they know, who can be named by their own names, representing their singularities in the environment in which they were immersed:

*“…left two years ago, was called M.L.”*

Calling the child by his name, talking about the qualities corresponds to taking him out of the condition of ‘thing’, and assigning marks that will make him a subject. There is an intertwining of the symbolic field with the imaginary field and the attribution of nicknames as affective games, made by social caregivers, helping in the insertion of the subject in the social environment:

*“[ … ] was called M.L. She had curly hair,”*

*“[ … ] today she asks to sit on my lap, raises her arms for us to take her.”*

As pointed out by social educators in their narratives, children arrive at the shelter with names assigned by their parents and, when this does not happen, the judges who do so. However, caregivers assign a new affectionate name, sometimes a part of their own names or a nickname. These often carry subjective attributes, marking affective places, as can be seen in the examples below:

*“[ … ] He is light [ … ] is an incredible child.”*

*“[ … ] very mischievous, used to run all around.”*

*“A.S. her name, that cutie [ … ].”*

### Axis 2: The Baby Caregiving Representation

It refers to the representation that the caregiver has about caring for the sheltered baby. Some themes were relevant:

#### Continuity and Discontinuity in Bonding and Caring

It addresses the issue of discontinuity in both the bond and the care; alluding to the lack of maintenance of the bond with the child after the adoption:

*“I shouldn’t tell you because here they don’t let you keep in touch with the children after they leave, but I keep in touch with the family and monitor her growth.”*

*“When I was there, she was very happy, and when I wasn’t there she missed me, she realized.”*

*“It was a child I met here and I intend to take it with me for the rest of my life, not only her, but the others as well.”*

#### Maternal Function/Biological Mother - Heart Mother/Limits of Caring

This theme is about the maternal function, pointing out the search for differentiation between the roles of mothers and caregivers:

*“R. needs a mother who doesn’t hurt her. We are not mothers, and there are always training courses here about it, but we always form a bond, even if it is forbidden.”*

*“For me being a mother is everything, I do everything for them. It is to abdicate a little of yourself to give to the child. First comes the child, then, me.”*

*“I’ve already been a heart mother of many children, all here. They need affection, care, but I know I’m not a real mother.”*

*“I think she thought that I was her mother and that was good, having someone she could trust, who protected her, made her feel safe.”*

*“Maternity is where you discover the other, now you deeply worry about another.”*

*“When it is your child, it’s different, the attention can be all his, here there are too many, it needs to be shared. It is quite different when you’re a mother. To me, being a mother is everything, I’ll do anything for them.”*

*“I feel love, but I know that son I only have two, the ones in the shelter are not mine, I feel love for them, but I want them to leave.”*

*“At the shelter, we take care of them, but with a family it is better, they receive more love.”*

It was evident in the participants’ narratives the difficulty of differentiating the role of caregiver and mother caregiver.

#### Frustration in Caring - Ability to Care for the Baby/Ability to Be With the Baby

In this theme, it is noted the frustration of caregivers for not being able to take care of babies as they would like:

*“I think she would like a family very much, a home, and she misses that, because here we cannot give all the love and attention they need, they’re so many.”*

The issue of dedicating themselves to the babies is demonstrated here. A feeling of guilt from the caregivers comes to light, as they are not able to offer all the babies the special attention, they assume they need:

*“If he were always in a family, it would be totally different, I see it with my children, the care I gave to each of them, each one needs a different care, attention.”*

#### Dyad of Caring/Group/Institutional/Social

The theme deals with the specificities of caring in the dyad, in the group, in the institution and in the social:

*“To be a social caregiver, it takes more than liking a child.”*

*“Sometimes people don’t believe in our work, but it is important.”*

*“Being an educator is having responsibility, it is having love, but we also suffer from the stories, but it is gratifying to see the development [ … ].”*

It was observed that when the social caregivers have time, they seek to give more attention and affection, as can be seen in the report on the importance of caring for babies affectionately:

*“[ … ] I just believe that they need affection and when there is time we need to give love, care.”*

The social caregivers who point to the family as the best option to leave the shelter, are actually referring to the extended family, that is, family members who can offer adequate support and care to the children, however, there are cases in which there is no family structure and this coexistence is impossible, leading then to the placement process in a substitute family:

*“Children should be with their families, and that they should bear the responsibilities and care.”*

*“I wish there was someone from the blood family to adopt.”*

Some reports indicated a preference for the child to be placed in a substitute family:

*“[ … ] I thank God that here we didn’t have many cases in which the children returned to their biological families [ … ] if I were a judge, I would never allow that, because if the family did not want, did not care, there was no reason to return “.*

*“I can’t imagine him in the biological family [ … ].”*

### Axis 3: Representation of Separation and Suffering

The third axis refers to the representation of the process of separation, the suffering and about the trauma.

#### Survivor Baby/Rescued Baby

The theme refers to the issue of surviving babies who were rescued:

*“He arrived here very sick, had several hospitalizations, suffered a lot, and I cried a lot, I suffered with him.”*

*“She arrived at the shelter very weak, she couldn’t even move.”*

#### Suffering Expressed by the Body/Injured Body

The baby’s body is represented, in the participants’ narratives, as a body that bears the marks of separation and suffering:

*“She was abused … She arrived very sad She was crying a lot, she felt anguish, she rolled the mat asleep.”*

*“She is lying down because she was like that when she arrived, lying down.”*

*“She arrived here with a more relaxed look in the eyes, I think it was due to the various hospitalizations she had, to be left in the hospital as soon as she was born and to have spent a month in the hospital after her birth.”*

#### Mistreated Baby/Untouchable Baby

The theme reflects the difficulty of taking care of the supposedly neglected and/or mistreated babies:

*“She was abused, and I was afraid to touch her, because she could think it was happening again.”*

#### Repetition of Violence

This theme picture the reference to the risk of repetition of negative experiences that babies absorbed and, consequently, to the transgenerational transmission of suffering and trauma:

*“He will pass on to his children what he received from his parents, so he will not be loving, caring, because he did not receive it from them.”*

*“I feel profound sadness to see that they are abandoned here, but I end up getting used to seeing so many stories and it takes a lot of strength because the children are distressed, and this passes on to us.”*

## Discussion

This work attempted an approximation of the experience of those who have the mission of taking care of institutionalized babies. We analyzed the different forms of care provided by the professionals at the institution, with the aim of proposing new ways more adapted to the needs of the population in question.

Working with neglected and/or suffering babies can be impactful, to the point of causing not only the rejection of what these victimizations represent, but also triggering a paradox in caregivers, implying in overcaring or failing to care properly.

There was an insistence on naming babies, which can translate into a search to humanize them and make them subjects; failing to consider them only as victims and allowing them other possibilities of existence. The attribution of names to things and people refers the subject to the field of reality, that is, it marks his existence. Therefore, naming also has a function regarding the psychic constitution (56).

This process also makes it possible to have a differentiation between the subjects, since the name also occupies a privileged place in the symbolic field (57). It is about giving the child a place of existence, through which they will read about this mark that has been attributed to them; that is, the name carries a load of desire from the one who named it.

Caregivers consider that all babies deserve special care. And when they are unable to provide idealized care, they feel guilty.

It was possible to observe the presence of the feeling of loss and anguish of separation and approximation resulting from the care practices applied by the caregivers to the institutionalized children. The narratives of the professionals about the care offered by them to babies, as specific to the development of infants, denote the recognition that the team that works at the institution is important for the evolution of the institutionalized.

They also pointed out that for the healthy development you need to be inserted into a protective and loving family environment, as the shelter cannot offer more specific care, because they should be divided to the same time to many babies; as noted in the literature on institutional care (10) in relation to the high turnover of babies and employees, and the high number of children for few professionals.

Another important factor observed in the narratives was the child’s right to know about his story. The revelation of biological experiences, prior to adoption, is a child’s right. Even if the parents do not talk about the subject, Dolto (58) points out that “the unconscious knows, but if its true story is not put into words, the child’s symbolic life will be on insecure bases” (p.235).

In view of the unfavorable position and the negative view of the shelter as a space to foster child development, the caregivers consider that it is most appropriate to be inserted into a family environment that enables the formation and establishment of lasting bonds (11).

The new relationships that the caregivers establish with the babies open the potential for transformation to the scripts or scenarios of suffering that accompany the babies. As Lachal (41) considers, the caregiver or professional with the mission to care and protect the baby is their environment. In these meetings, positive bonds are established, which allow the retrace of new possibilities for the baby’s development.

However, the baby does not yet have the ability to communicate through words. Thus, the absence of a narrative discourse implies that caregivers have the ability to decentralize themselves in a more elaborate form of communication, requiring a certain unusual regression for an adult. This process requires being able to enter the baby’s world, of course, when he invites us, living with the baby’s point of view, with the experiences, perceptions, feelings and at the same time trying to know and overcome the psychic invasion that the baby’s suffering can raise in the environment in which he is living. As Lachal (41) explains, these moments of affective agreement do not depend exclusively on the mother’s or caregiver’s ability to regress, but also on an exchange, complicity and association between the baby and the adult caregiver.

One of the aspects found in the analyzed material is the fact that caregivers avoid establishing an attachment with the baby so that they do not have to experience the loss, absence and separation from the baby. This could indicate that there would be a sharing of the baby’s experiences, including traumatic experiences with caregivers (40). There is a dilution of this trauma which contributes to the reconstruction of the baby’s psychic, emotional and physical state. In this context, before arriving at the shelter, the child who has already traveled along the routes of his life, during relationships and early experiences, moments of traumatic suffering in his emerging scenario, can gradually enter into a process of exchanging and sharing these experiences with adults caregivers, diluting and unraveling this emerging scenario. In this way, it is possible to create care devices adapted to the good development of babies, through the adapted material built together with the caregiver-baby dyad and the group, including researchers and care professionals, with a view to an environment adapted for good development of the baby.

Before the baby moves from one stage of development to another, he goes through these moments of transition, in which the trauma experienced dramatically influences the stages of his development, and there may be a loss of confidence, where he will have reliving traumatic situations.

A central point of the shelter institution is that caregivers can influence the development of these stages of traumatic experience (early separation, neglect, suffering)—it would be as if they were changing these stages (given that they do not know the baby’s dossier). The way he is cared in this transition phase, between the “nonprotective” family and the foster family—“idealized family” can influence how the caregivers will revive the trauma together with the baby. It could be said that one of the functions of the caregivers would be to act as a “catalyst” of emotions, of undetermined and unnamed experiences that could leave both the baby and those who care for him, in a process of sideration inherent to the traumatic scenario that each one lives (imaginary and real).

During the procedure of drawings and stories, a symbolic encounter takes place between “the caregiver and the baby.” While the caregiver draws and cohabits the baby’s story, it is possible to reestablish a bond with the baby through which coexistence is dual and group. These are moments of coexistence between the adult caregiver and the baby where the potentials involving care and the way care is instituted circulate. It is as if there was a new impact, a new space and a new time for a new construction of the “new.”

When talking to caregivers, the idea of “flashback” can be considered. The fact of asking to draw any baby and they draw the institutionalized babies allows us to consider that the DS-T procedure becomes a potential space where the baby can be thought in another way. The baby who supposedly lived in situations of neglect, early separation and physical and emotional aggressions, can be narrated by the adult caregiver in a way that appears humanized, receives a name, care, and affection; ceasing to be that baby who had been reduced to being a victim of the inadequacies of the aggressive, negligent and nonprotective biological family environment.

The baby who arrives at the institution has a course of crisis, shock and rupture that ends up involving other protagonists, whose are not belonging of the family, such as the caregivers, judges, researchers to try to change the script’s destiny, of the traumatic emerging scenario. Each stage of the experience of the story told, between the caregivers and the baby; between caregivers and the institutional working group and between caregivers and researchers, allows sharing the baby, with what this little being represents and the baggage he carries as well as the scenario that emerges from his life and in his environment. As previously said, even without speaking he is able to share and communicate because in the process of living together and exchanging from a shared agreement, the adult imagines a story about the imagined baby.

It can be considered that the baby tells his suffering and his needs with his gaze, his yell, his cry, depositing and sharing this suffering, that the adult caregiver will assign meanings and, from that, respond to the baby. It is this process of sharing the trauma that eases the density and intensity of pain, both for the baby and the adult caregiver (41).

In this study, the participants spoke and drew the baby from the institution, exposing the life experience that this baby lived. It is noticed in the analysis of the results that they liked to talk and to be heard about that feeling of the “unspoken” of the babies, of the baby’s impossibility to speak, but at the same time he is able to express the cry for help, or maybe, they are able to decode the scenario of a helpless and institutionalized baby.

The bond with the adult caregiver allows to change the traumatic mark previously established with a nonprotective adult. The exchanges and the way of care conducted by the caregivers allow the reconstruction of the universe of these institutionalized babies. Some questions inspire us, among them:

- How to train caregivers not to be invaded by the baby’s trauma and to be able to take care of him, give a care good enough for him to continue development?
- How to establish a favorable bond with the baby and vice versa?

## Conclusion

Institutionalized children or those deprived of their families experience a rupture in the mother-child relationship. The narratives and the graphic material made it possible to verify the difficulties that permeate the context of the performance of these professionals, such as the high turnover of children entering and leaving, as well as employees, the difficulty of dealing with the babies leaving, that is, the bonding process and bond breaking and nonrecognition of the profession. Emphasizing, mainly, the anguish and suffering of the participants from the reasons that they believe lead the children to be institutionalized.

It was also evident, through the interpretation of the data obtained in the DS-T procedure, that there are disagreements regarding the professionals’ conception about the placement in a substitute family or family reintegration. Most participants consider the institutionalization to be important, as it is a protective measure for children in situations of vulnerability and risk due to neglect, early separation and mistreatment.

Another aspect present in the narratives were questions about the formation of bonds between social caregivers and babies, and also the prohibition to keep in touch after the children left the institution. Therefore, it is possible to verify that the work with institutionalized babies causes a series of personal, emotional and structural issues that can have impacts. Therefore, the caregiver as the child can experience the formation and rupture of bonds at different times, which can generate losses understood as inherent to the emerging scenario present in the trauma transmission process.

It was highlighted that the narratives and drawings allowed us to observe that many of the social educators, although recognizing that the institutionalized babies are not their children and that the professional performance scenario is marked by pain and suffering, they imagine one day being remembered affectionately by adults who the babies will become, for having played a maternal role in their lives during the institutionalization period, which indicates that in addition to professional recognition there is a need for a place of affection.

The difficulty of differentiating personal life from professional life, was part of the narrative of social caregivers and other professionals participating in the study who called themselves, in some drawing-stories, as “mothers of the institution.” If, on the one hand, the feeling of mothering with the institutionalized babies can generate confusion of roles and feelings of sadness when the baby leaves the institution; on the other hand, there is evidence of the establishment of bonds between the professionals and the babies, these bonds being fundamental for healthy child development.

The analysis of the data obtained favored the understanding that the high number of institutionalized babies to a few professionals is an issue that needs to be rethought, since social caregivers sometimes told about it in their stories, justifying not offering care with quality due the lack of time and not due to negligence or unwillingness.

In this sense, it is important that Brazilian public policies regarding institutional care are rethought both in terms of the number of people being institutionalized and in the sense of seeking interventions that allow listening to social caregivers, allowing them a space to elaborate their losses and anguish, which directly impact on the baby care.

The study allowed to understand the imaginary and fantasies of the professionals in relation to the institutionalized babies, and the magical solution of an idealized future of success, in which children overcome the marks and frustrations caused by abandonment in early childhood and living in an institution.

## Data Availability Statement

The datasets generated for this study are available on request to the corresponding authors.

## Ethics Statement

The studies involving human participants were reviewed and approved by the Ethical Committee at São Camilo University, PQ.66/2018; and Embodied Document of CEP CAAEE 95842818.2.0000.0062, document number 2.843.363. Written informed consent to participate in this study was provided by the participants’ legal guardian/next of kin.

## Author Contributions

RN: conception and design of the study, organized the database, wrote the sections of the manuscript, wrote the first draft of the manuscript, performed the statistical analysis, and revised the manuscript. MO: conception and design of the major research, rewrote and revised the manuscript. GB: conception and design of the major research, revised the manuscript. AL: wrote the first draft of the manuscript, wrote the sections of the manuscript. FG: wrote the first draft of the manuscript, wrote the sections of the manuscript. TB: conception and design of the study, organized the database, wrote the sections of the manuscript, performed the statistical analysis, and revised the manuscript. All the authors contributed to manuscript revision, read, and approved the submitted version

## Conflict of Interest

The authors declare that the research was conducted in the absence of any commercial or financial relationships that could be construed as a potential conflict of interest.
